# Supplementary material for: Qualitative and Quantitative Detection of Potentially Virulent Vibrio parahaemolyticus in Drinking Water and Commonly Consumed Aquatic Products by Loop-Mediated Isothermal Amplification
Source: Pathogens. 2021 Dec 22;11(1):10. doi: 10.3390/pathogens11010010 (PMC8781264; doi:10.3390/pathogens11010010)
Supplement: Supplementary file 1 [file pathogens-11-00010-s001.zip › pathogens-1495434-supplementary.pdf]

Table S1. Bacterial strains and media used in this study

Strain	Genotype	Source	Medium
V. parahaemolyticus ATCC17802	*opaR, vpadF, ureC, tlh*	ATCC, United States	TSB
V. parahaemolyticus B1-22	*opaR, vpadF, tlh*	LS-SHOU, China	TSB
V. parahaemolyticus B3-8	*opaR, vpadF, tlh*	LS-SHOU, China	TSB
V. parahaemolyticus B4-13	*opaR, vpadF, tlh*	LS-SHOU, China	TSB
V. parahaemolyticus B4-28	*opaR, vpadF, tlh*	LS-SHOU, China	TSB
V. parahaemolyticus B6-13	*opaR, tlh*	LS-SHOU, China	TSB
V. parahaemolyticus B7-16	*opaR, vpadF, tlh*	LS-SHOU, China	TSB
V. parahaemolyticus B9-31	*opaR, vpadF, tlh*	LS-SHOU, China	TSB
V. parahaemolyticus B9-42	*opaR, vpadF, tlh*	LS-SHOU, China	TSB
V. parahaemolyticus B10-61	*opaR, vpadF, tlh*	LS-SHOU, China	TSB
V. parahaemolyticus B11-3	*opaR, vpadF, tlh*	LS-SHOU, China	TSB
V. parahaemolyticus L5-1	*opaR, vpadF, tlh*	LS-SHOU, China	TSB
V. parahaemolyticus L7-7	*opaR, vpadF, tlh*	LS-SHOU, China	TSB
V. parahaemolyticus L7-45	*opaR, vpadF, tlh*	LS-SHOU, China	TSB
V. parahaemolyticus L10-15	*opaR, vpadF, tlh*	LS-SHOU, China	TSB
V. parahaemolyticus N2-8	*opaR, vpadF, tlh*	LS-SHOU, China	TSB
V. parahaemolyticus N2-11	*opaR, vpadF, tlh*	LS-SHOU, China	TSB
V. parahaemolyticus N2-20	*opaR, vpadF, tlh*	LS-SHOU, China	TSB
V. parahaemolyticus N2-25	*opaR, tlh*	LS-SHOU, China	TSB
V. parahaemolyticus N3-2	*opaR, vpadF, tlh*	LS-SHOU, China	TSB
*V. parahaemolyticus*N3-3	*opaR, vpadF, tlh*	LS-SHOU, China	TSB
V. parahaemolyticus N3-11	*opaR, vpadF, tlh*	LS-SHOU, China	TSB
V. parahaemolyticus N3-13	*opaR, vpadF, tlh*	LS-SHOU, China	TSB
V. parahaemolyticus N3-29	*opaR, vpadF, tlh*	LS-SHOU, China	TSB
V. parahaemolyticus N3-30	*opaR, vpadF, tlh*	LS-SHOU, China	TSB
V. parahaemolyticus N3-32	*opaR, vpadF, tlh*	LS-SHOU, China	TSB
V. parahaemolyticus N3-33	*opaR, tlh*	LS-SHOU, China	TSB
V. parahaemolyticus N4-9	*opaR, vpadF, tlh*	LS-SHOU, China	TSB
V. parahaemolyticus N4-26	*opaR, vpadF, tlh*	LS-SHOU, China	TSB
V. parahaemolyticus N4-31	*opaR, vpadF, tlh*	LS-SHOU, China	TSB
V. parahaemolyticus N4-46	*opaR, vpadF, tlh*	LS-SHOU, China	TSB
V. parahaemolyticus N5-15	*opaR, vpadF, tlh*	LS-SHOU, China	TSB
V. parahaemolyticus N6-7	*opaR, vpadF, tlh*	LS-SHOU, China	TSB
V. parahaemolyticus N6-10	*opaR, vpadF, tlh*	LS-SHOU, China	TSB
V. parahaemolyticus N6-16	*opaR, vpadF, tlh*	LS-SHOU, China	TSB
V. parahaemolyticus N6-26	*opaR, vpadF, tlh*	LS-SHOU, China	TSB
V. parahaemolyticus N7-3	*opaR, tlh*	LS-SHOU, China	TSB
V. parahaemolyticus N7-9	*opaR, vpadF, tlh*	LS-SHOU, China	TSB
V. parahaemolyticus N7-45	*opaR, vpadF, tlh*	LS-SHOU, China	TSB
V. parahaemolyticus N7-69	*opaR, vpadF, tlh*	LS-SHOU, China	TSB
V. parahaemolyticus N8-9	*opaR, vpadF, tlh*	LS-SHOU, China	TSB
V. parahaemolyticus N8-13	*opaR, vpadF, tlh*	LS-SHOU, China	TSB
V. parahaemolyticus N8-35	*opaR, vpadF, tlh*	LS-SHOU, China	TSB
V. parahaemolyticus N9-24	*opaR, vpadF, tlh*	LS-SHOU, China	TSB
V. parahaemolyticus N9-31	*opaR, vpadF, tlh*	LS-SHOU, China	TSB
V. parahaemolyticus N10-20	*opaR, vpadF, tlh*	LS-SHOU, China	TSB
V. parahaemolyticus N10-48	*opaR, tlh*	LS-SHOU, China	TSB
V. parahaemolyticus Q5-6	*opaR, vpadF, tlh*	LS-SHOU, China	TSB
V. parahaemolyticus Q8-2	*opaR, vpadF*	LS-SHOU, China	TSB
V. parahaemolyticus Q8-7	*opaR, vpadF, tlh*	LS-SHOU, China	TSB
V. parahaemolyticus Q8-15	*opaR, vpadF, tlh*	LS-SHOU, China	TSB
Vibrio alginolyticus ATCC17749	-	ATCC, United States	TSB
Vibrio alginolyticus ATCC33787	-	ATCC, United States	TSB
Vibrio fluvialis ATCC33809	-	ATCC, United States	Marine 2216
Vibrio harvey ATCC BAA-1117	-	ATCC, United States	Marine 2216

Vibrio harvey ATCC33842	-	ATCC, United States	Marine 2216
Vibrio metschnikovii ATCC 700040	-	ATCC, United States	Marine 2216
Vibrio mimicus bio-56759	-	Biobw, China	TSB
Vibrio vulnificus ATCC27562	-	ATCC, United States	TSB
Vibrio vulnificus	-	DL, China	LB
Aeromonas hydrophila ATCC35654	-	ATCC, United States	TSB
Aeromonas hydrophila	-	-	LB
Enterobacter cloacae ATCC13047	-	ATCC, United States	TSB
Enterobacter cloacae	-	SJAM, China	LB
Escherichia coli ATCC8739	-	ATCC, United States	TSB
Escherichia coli ATCC25922	-	ATCC, United States	LB
Escherichia coli K12	-	IIM, China	TSB
Enterobacter sakazakii CMCC45401	-	Biobw, China	TSB
Klebsiella oxytoca 0707-27	-	LS-SHOU, China	EE
Klebsiella pneumoniae 0717-1	-	LS-SHOU, China	EE
Klebsiella pneumoniae 1202	-	LS-SHOU, China	EE
Klebsiella variicola 0710-01	-	LS-SHOU, China	EE
Lactobacillus casei D31	-	LS-SHOU, China	MRS
Lactobacillus casei T9	-	LS-SHOU, China	MRS
Lactobacillus casei K17	-	LS-SHOU, China	MRS
Listeria monocytogenes ATCC19115	-	ATCC, United States	BHI
Pseudomonas aeruginosa ATCC9027	-	ATCC, United States	TSB
Pseudomonas aeruginosa ATCC27853	-	ATCC, United States	TSB
Salmonella enterica subsp. Enterica-Leminor et popoff ATCC13312	-	ATCC, United States	TSB
Staphylococcus aureus ATCC 25923	-	ATCC, United States	TSB
Staphylococcus aureus ATCC 8095	-	ATCC, United States	TSB
Staphylococcus aureus ATCC29213	-	ATCC, United States	TSB
Staphylococcus aureus ATCC6538	-	ATCC, United States	TSB
Staphylococcus aureus ATCC6538P	-	ATCC, United States	TSB
Shigella dysenteriae CMCC51252	-	ATCC, United States	TSB
Salmonella spp.	-	-	TSB
Shigella flexneri CMCC51572	-	GCCC, China	TSB
Shigella flexneri ATCC12022	-	ATCC, United States	TSB
Shigella flexneri CMCC51574	-	GCCC, China	TSB
Salmonella paratyphi-ACMCC50093	-	GCCC, China	TSB
Shigella sonnei ATCC25931	-	ATCC, United States	TSB
Shigella sonnet CMCC51592	-	GCCC, China	TSB
Salmonella typhimurium ATCC15611	-	ATCC, United States	TSB
Staphylococcus aureus	-	-	LB
V.cholerae ATCC39315	-	ATCC, United States	*
V.cholerae GIM1.449	-	GCCC, China	TSB
V.cholerae 805-38	-	LS-SHOU, China	TSB
V.cholerae 717-01	-	LS-SHOU, China	TSB
V.cholerae 805-29	-	LS-SHOU, China	TSB
V.cholerae 805-32	-	LS-SHOU, China	TSB
V.cholerae 717-25	-	LS-SHOU, China	TSB

*, genomic DNA available. −, unknown. ATCC: American Type Culture Collection, United States; DL: Dishui Lake, Shanghai, China; GCCC, Guangdong Culture Collection Center, Guangzhou, China; IIM, Institute of Industrial Microbiology, Shanghai, China; LS-SHOU, Laboratory stock, Shanghai Ocean University, Shanghai, China; SJAM, Shanghai Jiangyang Aquatic Market, Shanghai, China; Virulence-associated gene : *opaR*, *vpadF*, *ureC*, and *tlh*.

Table S2. Target gene sequences in some representative *V. parahaemolyticus* strains used in this study.

V. Parahemolyticus strain	Gene	NCBI accession number	*V. Parahemolyticus* strain	Gene	NCBI accession number
B1-22	*opaR*	MZ819080	N4-26	*tlh*	OL409136
B3-8	*opaR*	OK490369	N7-19	*tlh*	OL409137
B4-13	*opaR*	OL362198	N8-9	*tlh*	OL409138
B4-28	*opaR*	OL362204	N8-13	*tlh*	OL409139
B7-16	*opaR*	OL362205	N8-36	*tlh*	OL409140
B9-42	*opaR*	OL362199	N4-31	*tlh*	OL409141
B10-61	*opaR*	OL362206	N7-3	*tlh*	OL409142
B11-3	*opaR*	OL362200	N7-9	*tlh*	OL409143
L7-45	*opaR*	OL362201	N7-45	*tlh*	OL409144
L10-15	*opaR*	OL362202	N9-24	*tlh*	OL409145
N2-8	*opaR*	OL362207	N9-31	*tlh*	OL409146
N2-11	*opaR*	OL362203	N10-20	*tlh*	OL409147
N2-20	*opaR*	OL422814	N10-48	*tlh*	OL409148
N3-13	*opaR*	OL422815	B4-28	*vpadF*	OL409149
N3-32	*opaR*	OL422816	N4-9	*vpadF*	OL409150
N3-33	*opaR*	OL422817	N7-9	*vpadF*	OL409151
N6-10	*opaR*	OL422818	L5-1	*vpadF*	OL409152
N9-31	*opaR*	OL422819	L7-7	*vpadF*	OL409153
Q5-6	*opaR*	OL422820	N3-2	*vpadF*	OL409154
Q8-15	*opaR*	OL422821	N3-13	*vpadF*	OL409155
B6-13	*tlh*	OL409129	N3-30	*vpadF*	OL409156
L7-7	*tlh*	OL409130	N4-31	*vpadF*	OL409157
L7-45	*tlh*	OL409131	N4-46	*vpadF*	OL409158
N2-25	*tlh*	OL409132	N6-26	*vpadF*	OL409159
N3-2	*tlh*	OL409133	N7-19	*vpadF*	OL409160
N3-3	*tlh*	OL409134	ATCC17802	*ureC*	CP014047.2
N4-9	*tlh*	OL409135			
